# Nucleated red blood cells: Immune cell mediators of the antiviral response

**DOI:** 10.1371/journal.ppat.1006910

**Published:** 2018-04-26

**Authors:** Ivan Nombela, Maria del Mar Ortega-Villaizan

**Affiliations:** IBMC, Miguel Hernández University, Elche, Spain; University of Michigan Medical School, UNITED STATES

## Introduction

The involvement of nucleated red blood cells (RBCs) as immune response cell mediators is a novel topic of research. RBCs are the most abundant cell type in the bloodstream and are best known for their roles in gas exchange and respiration. In mammals, mature RBCs are flexible, oval, biconcave disks that lack cell nuclei, organelles, and ribosomes (reviewed in Moras et al. 2017 [1]). In nonmammalian vertebrates, RBCs are oval, flattened, biconvex disks with a cytoskeleton composed of a marginal band of microtubules and a cell nucleus and organelles in their cytoplasm [2], which allow them to de novo synthesize proteins and molecules in response to stress and stimuli. In the recent past, a set of biological processes related to immunity–such as phagocytosis [3], antigen presentation [3], and interleukin-like production [4–7]–have been reported in nucleated RBCs from different species. However, elucidating the role of RBCs during viral infections is an emergent research topic of great interest. Here, we provide a brief overview of the novel role of nucleated RBCs against viral infections.

### Viral pathogen-associated molecular patterns (PAMPs) induce pattern-recognition receptor (PRR) signaling in nucleated RBCs

Nucleated RBCs are implicated in the immune response to viral infections based on their response to viral PAMPs through various PRR signaling pathways. Among these receptors, the expression of Toll-like receptor 3 (TLR3) and TLR9–which are endosomal TLRs that recognize viral double-stranded RNA (dsRNA) and nonmethylated viral 5'-C-phosphate-G-3' (CpG)-containing DNA, respectively–and retinoic acid-inducible gene I (RIG-I)–a member of the RIG-I-like receptor (RLR) family that interacts intracellularly with viral dsRNA–have been reported in rainbow trout RBCs [5, 8] and Atlantic salmon [9], respectively. Chicken RBCs constitutively express *tlr3* and *tlr21*, which is a homolog of mammalian TLR9 [5, 10]. Stimulation of these receptors with their corresponding PAMPs leads to the activation of signaling networks that induce the transcription of a set of genes, resulting in a characteristic immune response.

The activation of these receptors by viral pathogens induces expression of the interferon system [11, 12]. Stimulation of rainbow trout RBCs with polyinosinic:polycytidylic acid (poly I:C, a molecule structurally similar to dsRNA) induces the de novo synthesis of mRNAs from immune genes such as chemokine (C-C motif) ligand 4 (*ccl4*), interferon-α (*ifn-α*), and myxovirus resistance gene *(mx)* [5]; and in chickens, RBCs respond to poly I:C by upregulating type I IFN (*ifn1*) and interleukin-8 (*il-8*) genes [10]. Moreover, the infectious pancreatic necrosis virus (IPNV)–a dsRNA virus–has been reported to stimulate the expression of *tlr3*, *ifn1* and *mx* genes [13]. The piscine orthoreovirus (PRV) also increases the expression of *rig-I*, *mx*, and *ifn-α* genes in Atlantic salmon RBCs [6].

The roles that other members of the RLR family, such as melanoma differentiation-associated protein 5 (MDA5) or probable ATP-dependent RNA helicase DExH-box helicase 58 (LGP2), assume in RBCs are still unknown. In addition, we still do not know if RBCs express other PRRs that recognize viral genomic RNA, such as TLR7 or TLR8. While IFN1 is thought to play a similar role in mammalian and nonmammalian species and induce similar sets of genes [14], the extent of nucleated RBCs’ involvement in the global organism IFN1 response and how RBCs’ involvement influences defense against viral infections remain to be defined.

### Nucleated RBCs may be capable of inducing an adaptive immune response

Nucleated RBCs are linked to the adaptive immune response. Major histocompatibility complex I (MHCI) plays a key role in the antigen presentation of intracellular pathogens, which initiates adaptive immunity mechanisms. MHCI is expressed on the surface of RBCs from rainbow trout [15], Atlantic salmon [6], African clawed frogs [16], and chickens [17]. However, to date, it has only been reported that PRV infection induces genes involved in antigen presentation via MHCI in salmon RBCs [6] and that poly I:C upregulates gene ontology (GO) categories related to antigen processing, antigen presentation, and MHCI receptor activity in rainbow trout RBCs [18].

Molecules bearing the immunoreceptor tyrosine-based activation motif (ITAM), which is contained in certain transmembrane proteins of the immune system and is important for signal transduction in immune cells, are known markers of hematopoietic and immune cells [19]. ITAM-bearing molecules are expressed on rainbow trout RBCs [20]. Further, Epstein–Barr virus G-protein-coupled receptor 2 (EBI2) plays a critical role in the regulation of T cell–dependent antibody responses and provides a mechanism to balance short- versus long-term antibody responses [21]. EBI2 is highly expressed in rainbow trout young RBCs [22]. The presence of these molecules in nucleated RBCs may indicate a role of these cells in the adaptive immune response. However, the function of these molecules on RBCs and their effect on the antiviral adaptive immune response remain to be studied.

### Nucleated RBCs trigger diverse immune responses against viral aggression

**Fig 1:**
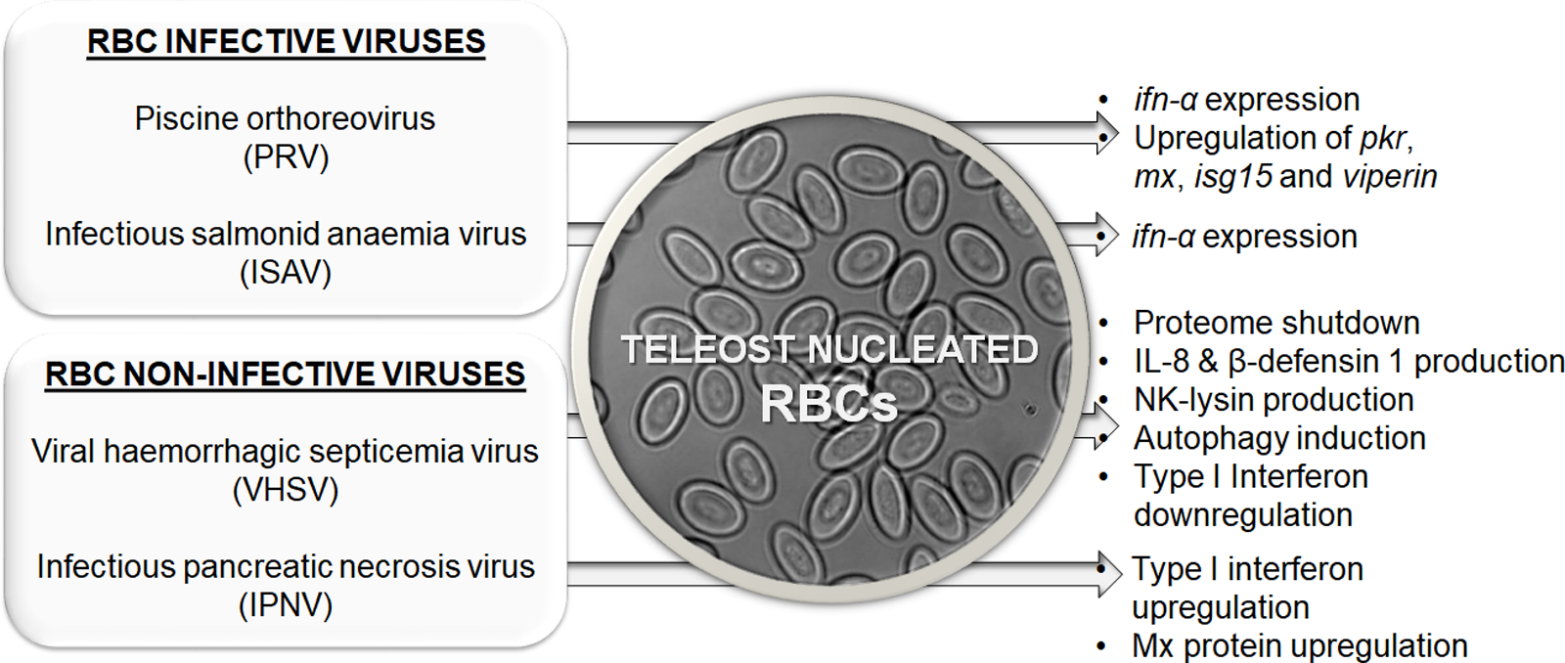
Schematic representation of teleost nucleated RBC immune responses against different infective (target: RBCs) or noninfective (target: other cell types) viral pathogens. *ifn-α*, interferon-α; IL-8, interleukin-8; *isg15*, interferon-stimulated gene 15; *mx*, myxovirus resistance gene; *pkr*, protein kinase RNA-activated; RBC, red blood cell.

Three viruses from different families that infect or replicate inside nucleated RBCs have been identified: (i) infectious salmonid anemia virus (ISAV) from the Orthomyxoviridae family with single-stranded RNA (ssRNA) [7], (ii) PRV from the Reoviridae family with dsRNA [6, 23], and (iii) erythrocytic viral infections, reviewed in Paperna and Alves de Matos [24]. Fig 1 schematically summarizes the response of nucleated RBCs to these viruses. Unfortunately, information on the immune response of RBCs to erythrocytic viral infections is not available.

A study of nucleated RBCs from ISAV-infected Atlantic salmon first demonstrated the ability of RBCs to induce an immunological response against a viral pathogen. This response was characterized by the induction of *ifn-α* in hemagglutinated RBCs [7]. Recently, it has been shown that PRV also can induce the expression of *ifn-α*–in addition to *mx*, protein kinase RNA-activated (*pkr*) [6], *viperin*, and interferon-stimulated gene 15 (*isg15*) [25] antiviral genes–in PRV-challenged Atlantic salmon RBCs.

**Fig 2:**
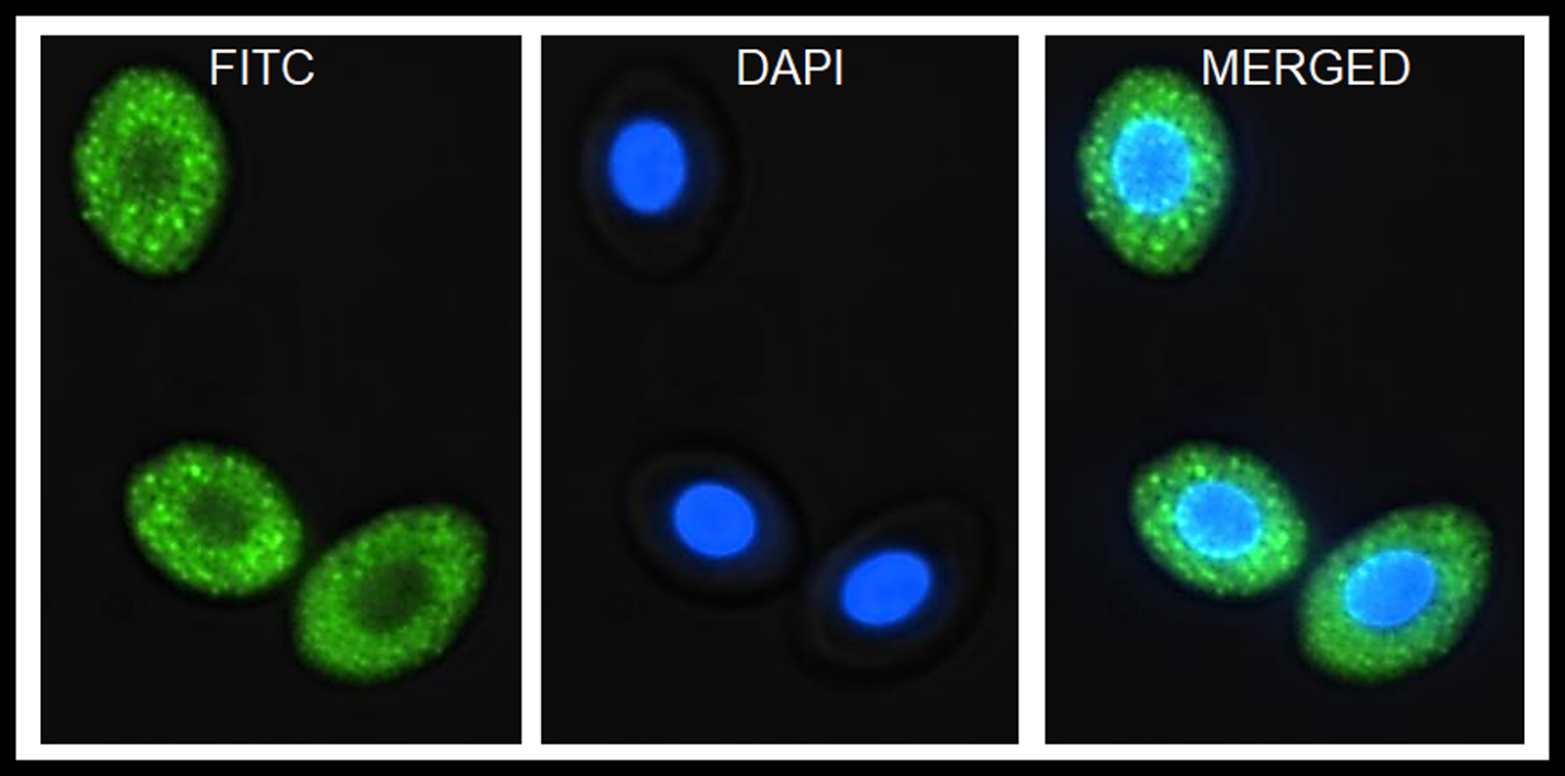
Constitutive expression of Mx antiviral protein in rainbow trout nucleated RBCs. Immunofluorescence images of Mx protein expression in nucleated RBCs. FITC: Mx protein expression; DAPI: nuclei. Images were obtained using an INCell Analyzer 6000 Cell imaging system (GE Healthcare, Little Chalfont, United Kingdom). DAPI, 4’,6-diamidino-2-phenylindole; FITC, Fluorescein-5-isothiocyanate; Mx, myxovirus resistance gene; RBC, red blood cells.

Recently, Nombela and colleagues demonstrated that nucleated RBCs can generate immune responses to viruses despite not being infected. Rainbow trout RBCs are nonpermissive to viral hemorrhagic septicemia virus (VHSV) [26] and infectious pancreatic necrosis virus (IPNV) infections [13], likely due to the inability of VHSV and IPNV to replicate in ex vivo purified rainbow trout RBCs. This phenomenon is known as nonproductive or abortive infection in nonpermissive cells and occurs when a virus enters a host cell and some or all viral components are synthesized but nonproductive or defective viruses are ultimately released because the host cell is nonpermissive or inhibits the replication of the virus. Previously, abortive infection in a macrophage cell line was linked with the constitutive expression of the antiviral Mx protein by macrophages [27]. Similarly, high levels of constitutive Mx transcripts and protein have been identified in rainbow trout RBCs (Fig 2), suggesting a possible mechanism for aborted or halted infections in RBCs [13, 26]. Nevertheless, rainbow trout RBCs can develop diverse immune responses to VHSV halted replication, a process characterized by global proteome downregulation–mainly of proteins from the proteasome and RNA stability processes–increased expression of IL-8 and β-defensin 1, decreased expression of genes related to the IFN1 pathway, and an antioxidant response [13]. In the case of IPNV aborted infection in rainbow trout RBCs, there was an increase in the expression of *ifn1*, *mx*, interferon regulatory factor 7 (*irf7*), and *pkr* genes, followed by upregulation of Mx protein expression [13] (summarized in Fig 1).

Considering their ability to produce immune proteins related to interferon, pro-inflammatory cytokines, antimicrobial peptides, proteasome [26], and autophagy [28] pathways, nucleated RBCs likely are able to trigger an immune response similar to that of their leukocyte counterparts by activating diverse immune mechanisms to complement the protection against infection conferred to the host organism.

### Nucleated RBCs can mount immune responses against nonviral pathogens

The RBCs of mammalian and nonmammalian vertebrates are hosts for approximately 40 genera, including protists, prokaryotes, and viruses [18, 29]. Few blood infections of fish, amphibians, reptiles, and birds have proven pathogenicity, in contrast to the many known intraerythrocytic mammalian pathogens [29]. To date, few studies have reported diverse immune responses of nucleated RBCs to this broad spectrum of pathogens.

As previously described, the immune response against viruses is generally associated with the expression of IFN1 and ISGs. In response to bacterial lipopolysaccharide (LPS), rainbow trout RBCs upregulate the expression of tumor necrosis factor receptor-like (*tnfr-like*), oxidative-stress response 1 (*oxsr1*), *irf1*, and *mhcI* genes. Several reports have shown that hemoglobin, the most abundant protein of RBCs, has antibacterial activity and can elicit antimicrobial activity through reactive oxygen species production when under pathogen attack [30]. In rainbow trout, acid-soluble extracts from RBCs showed antibacterial activity against a variety of bacteria, including *Planococcus citreus* and *Escherichia coli* [31]. In the presence of the fungus *Candida albicans*, rainbow trout [3], and chickens [4], RBCs performed innate immunity functions, using phagocytosis to bind and engulf *C*. *albicans* and present to macrophages. Ultimately, little is known regarding the immune response triggered by nucleated RBCs against the broad range of pathogens that infect them.

### Nucleated RBCs are future targets for vaccines

Human non-nucleated RBCs have long been investigated for the transportation of drugs or antigens through the blood [32, 33]. Proteomic studies of human [34] and nonhuman primate species [35] aim to further characterize the biology of human RBCs and identify future targets for newer-generation vaccines, especially against malaria. Because of the ability of nucleated RBCs to generate and modulate immune responses, development of a new generation of vaccines targeting membrane receptors or intracellular molecules of nucleated RBCs capable of triggering and stimulating the antiviral immune response is a promising and exciting field. Such vaccines may contribute greatly to organism survival, given the large volume of RBCs and their fast distribution through the organism. However, additional proteomic studies of nucleated RBCs are needed to identify potential therapeutic targets.
